# Leptin promotes epithelial-mesenchymal transition of breast cancer via the upregulation of pyruvate kinase M2

**DOI:** 10.1186/s13046-016-0446-4

**Published:** 2016-10-21

**Authors:** Lan Wei, Kuangfa Li, Xueli Pang, Bianqin Guo, Min Su, Yunxiu Huang, Nian Wang, Feihu Ji, Changli Zhong, Junhong Yang, Zhiqian Zhang, Yulin Jiang, Yifeng Liu, Tingmei Chen

**Affiliations:** 1 Key Laboratory of Diagnostic Medicine Designated by the Ministry of Education, Chongqing Medical University, Chongqing, 400016 China; 2 Department of Clinical Laboratory, Chongqing Cancer Institute, Chongqing, 400030 China

**Keywords:** Breast cancer, Leptin, Epithelial-mesenchymal transition, Pyruvate kinase M2

## Abstract

**Background:**

Accumulating researches have shown that epithelial-mesenchymal transition (EMT) contributes to tumor metastasis. Leptin, a key adipokine secreted from adipocytes, shapes the tumor microenvironment, potentiates the migration of breast cancer cells and angiogenesis, and is also involved in EMT. However, the potential mechanism remains unknown. This study aims to explore the effect of leptin on EMT in breast cancer cells and the underlying mechanism.

**Methods:**

With the assessment of EMT-associated marker expression in MCF-7, SK-BR-3, and MDA-MB-468 cells, the effect of leptin on breast cancer cells was analyzed. Besides, an array of pathway inhibitors as well as RNA interference targeting pyruvate kinase M2 (PKM2) were used to clarify the underlying mechanism of leptin-mediated EMT in vitro and in vivo.

**Results:**

The results demonstrated that leptin promoted breast cancer cells EMT, visibly activated the PI3K/AKT signaling pathway, and upregulated PKM2 expression. An antibody against the leptin receptor (anti-ObR) and the PI3K/AKT signaling pathway inhibitor LY294002 significantly abolished leptin-induced PKM2 expression and EMT-associated marker expression. SiRNA targeting PKM2 partially abolished leptin-induced migration, invasion, and EMT-associated marker expression. In vivo xenograft experiments indicated that RNA interference against PKM2 suppressed breast cancer growth and metastasis.

**Conclusions:**

Our data suggest that leptin promotes EMT in breast cancer cells via the upregulation of PKM2 expression as well as activation of PI3K/AKT signaling pathway, and PKM2 might be one of the key points and potential targets for breast cancer therapy.

## Background

Breast cancer is a leading cause of cancer death in women [1]. Accumulating evidence has shown that obesity is a significant risk and negative prognosis factor for breast cancer [2]. Adipose tissue plays a crucial role as an energy storage depot, and currently, there is clear evidence that adipocytes act as endocrine cells and produce various biologically active substances, such as growth factors, cytokines, adipokines and leptin [3, 4]. In spite of the strongly suggested association between obesity and breast cancer, the underlying molecular mechanisms are not known clearly. Previous studies indicate that leptin, one of the adipokines secreted from adipocytes, is an important factor that links obesity with breast cancer [1, 5].

Leptin, a 16-kDa single-chain proteohormone encoded by the *LEP* gene, is expressed in a variety of tissues, including placenta, ovaries, mammary epithelium, bone marrow, and lymphoid tissues [6–9]. Leptin acts through specific leptin receptors (ObRs) and is a key factor in controlling the biological effects of food intake, energy balance, immune, and endocrine systems, as well as ontogenesis. Most of these functions are involved in leptin-induced signals which comprise several pathways triggered by many cytokines (i.e., canonical signaling pathways: JAK2/STAT, MAPK/ERK, and PI3K/AKT kinase) [10]. Leptin/ObRs are expressed at low levels in the epithelial cells of normal human mammary glands, but overexpressed in breast cancer cells [9, 11, 12].

Recent studies have shown that EMT is a crucial initiator of and a contributor to tumor invasion and migration [13]. During EMT, cancer cells undergo morphological changes, such as cell-cell junction dissolution, loss of apical-basolateral cell polarity, and acquisition of mesenchymal marker expression [14]. Snail, Twist and ZEB, and the important transcription factors of EMT are critical points in the study the mechanism of EMT. Twist is a highly conserved transcription factor and involves in organ development, cell proliferation, differentiation and tumorigenesis [15, 16], and it is also a major regulator in EMT and promotes tumor invasion and metastasis [17–19]. Our previous study showed that leptin and interleukin 8(IL-8) induced EMT in breast cancer cells via the PI3K/AKT signal pathway [20], and this signal pathway was a significant canonical signaling pathway in leptin-induced signals. Besides IL-8 which is involved in leptin-induced EMT, this study has found that PKM2 is another critical molecule affecting tumor progression.

Pyruvate kinase (PK) participates in the final rate-limiting step of glycolysis and catalyzes phosphoenolpyruvate(PEP) and ADP to pyruvate and ATP [21]. PKM1, PKM2, PKL and PKR are four isoforms of PK, and they are expressed in different types of mammalian cells and tissues [22]. PKM2 is expressed during embryonic development, but it is absent from most adult tissues [23]. There are reports indicating that PKM2 is overexpressed in malignant cells and plays the central role not only in metabolic reprogramming but also in directed regulation of tumor progression, and PKM2 could promote EMT in colorectal cancer and hepatocellular carcinoma [14, 24, 25].

In this study, the role of PKM2 in leptin-induced EMT in breast cancer cells is investigated; it is suggested that leptin promoted EMT in breast cancer cells via the upregulation of PKM2 expression as well as activation of PI3K/AKT signaling pathway, and PKM2 might be one of the key points and potential targets for breast cancer therapy.

## Methods

### Cell culture

The human breast cancer cell lines MCF-7, SK-BR-3 and MDA-MB-468 were obtained from American Type Culture Collection and maintained in DMEM supplemented with 10 % fetal bovine serum (FBS, Gibco). The cells were cultured at 37 °C in a humidified incubator with 5 % CO_2_.

### Immunofluorescence analysis

MCF-7, SK-BR-3 and MDA-MB-468 cells were grown on coverslips. Cells were washed with PBS, fixed with 4 % paraformaldehyde at room temperature for 20 min, permeabilized with 0.3 % Triton X-100, and blocked with 5 % goat serum for 30 min. All cells were incubated overnight at 4 °C with the corresponding primary antibodies(OBR mouse anti-human) in blocking solution, washed three times with PBS, and incubated for 1 h in darkness at room temperature with secondary antibodies (TRITC-conjugated goat anti-mouse). After washing, nuclei were stained with DAPI for 10 min in darkness at room temperature, and then washed three times with PBS and the coverslips were mounted with 95 % glycerol. Cell fluorescence was examined using a fluorescence microscopy (Olympus, Japan).

### RNA isolation and quantitative real-time (qRT)-PCR Assay

Total RNA was isolated using Trizol reagent (TAKARA, Japan) according to the manufacturer’s protocol. RNA was stored at −80 °C after being eluted with RNase-free water. RNA concentrations were measured by spectrophotometer and RNA was reversely transcribed to cDNA using reverse transcription kit (TAKARA, Japan). Real-time (RT)-PCR was implemented using iTaq Universal SYBR Green One-step kit (BioRad). Results were normalized to the endogenous β-actin mRNA. The following primers were used: PKM2 sense 5’-CCATCCTCTACCGGCCCGTTG’, PKM2 antisense 5’- CCAGCCACAGGATGTTCTCGTC-3’, β-actin sense 5’-CCTTCCTGGGCATGGAGTCCT-3’, β-actin sense 5’-CCTTCCTGGGCATGGAGTCCT-3’, β-actin antisense 5’- GGAGCAATGATCTTGATCTTC-3.

### Cell transfection and infection

MCF-7 and SK-BR-3 were placed in 6-well plates at 2 × 10^5^ per well. Twenty-four hours after placing, siRNA against PKM2 and negative miRNA (GenePharma, Shanghai) were transfected to the cells using lipofectamine 2000 liposomes (Invitrogen) according to the manufacturer’s protocol. After culturing for 48 h, transfection efficiency was quantified by Western blotting. The sequences of siRNAs were as follows: PKM2 sense 5’- CAUCUACCACUUGCAAUUATT-3’, anti-sense 5’- UAAUUGCAAGUGGUAGAUGTT-3’; negative control sense 5’-UUCUCCGAACGUGUACGUTT’, anti-sense 5’- ACGUGACACGUUCGGAGAATT-3’. Lentivirus-based short hairpin RNA (shRNA) vector and lentivirus-based cDNA targeting the PKM2 gene were constructed by GenePharma (Shanghai, China). PKM2 shRNA was generated with CATCTACCACTTGCAATTA oligonucleotide targeting exon 10 of the PKM2 transcript. Lentiviru vector shRNA was generated with CATCTACCACTTGCAATTA oligonucleotide. Cells were selected with puromycin for 14 days at 37 °C after being infected with lentiviral. The effectiveness of transfection using lentiviruses in SK-BR-3 cells was verified by Western blotting.

### Western blot

All proteins from cancer cells were subjected to SDS-PAGE and then transferred to PVDF membrane. Blots were probed with PKM2, p-PKM2, p-AKT (Ser473), AKT antibody (Cell Signaling Technologies, USA), OBR antibody (Santa, USA), E-cadherin, vimentin, and fibronectin antibody (Bioword, USA). Membranes were analyzed using Enhanced Chemiluminescence (ECL) detection system (VIAGENE, USA).

### Wound healing migration and matrigel invasion assays

For wound healing migration assays, cells were seeded onto 6-well plates. After treatment, the cell monolayer was scratched with a pipette tip and then washed three times to remove the floating cells. Then, fresh serum-free medium with leptin was added, and photos were taken at 0, 24, 36 and 72 h using a microscope (Olympus, Japan). Scratch areas were measured using image software. For invasion assays, tumor cells were performed using transwell system (Millipore, USA) with 8 μm-pore polycarbonate filter membrane. The chambers were pre-coated with 50 μl matrigel (1:7 dilution; Sigma, USA). The upper chambers were seeded with 2 × 10^4^ tumor cells in serum-free medium and the lower chambers were filled with medium containing 15 % fetal bovine serum as a chemo-attractant. After being incubated for 24 h, cells on the interior of upper chamber were scrubbed and the invading cells in the lower chambers were fixed with 4 % paraformaldehyde. Then, the polycarbonate membranes were stained with 0.1 % crystal violet for 10 min at room temperature. The number of invasion cells in five randomly selected fields under microscope (Olympus, Japan) was counted.

### Xenografts of nude mice

Breast cancer cells (5 × 10^6^/per inoculation) were injected into mammary fat pads of 5-week-old female nude mice (*n* = 9/group). Tumor volumes were measured using calipers, and defined as ab^2^/2(a: length, b: width). Mice were sacrificed when 5 weeks of post-injection, and tumors and lungs were removed, fixed in 4 % formaldehyde, and emdedded in paraffin. Tumor formation and proteins were detected by H&E staining and immunohistochemical staining. This study was approved by the Ethical Committee of Chongqing Medical University.

### Immunohistochemistry

The expression of E-cadherin, vimentin, fibronectin, Twist and PKM2 in xenografts of nude mice were determined using immunohistochemistry. After being deparaffinized, tissue paraffin sections were heat-treated with citrate buffer (0.01 mol/pH6.0) as an epitope retrieval protocol. Endogenous peroxidase was blocked with 3 % H_2_O_2_ for 15 min, followed by rinsing twice, and then sections were incubated with 5 % goat serum for 30 min to avoid non-specific binding. After that, sections were incubated with PKM2 antibody (Cell Signaling Technologies, USA), E-cadherin, vimentin and fibronectin antibody (Bioword, USA) (1:200 dilution; 5 % BSA) at 4 °C overnight. Subsequently, sections were washed with PBS 5 times, and incubated with HRP-conjugated secondary antibody (BiYunTian, China) for 1 h. The color was developed using 3-3’-diaminobenzidine after being washed 5 times with PBS. Counterstaining was performed with hematoxylin. Finally, sections were dehydrated and mounted with a neutral resin.

### Statistical analysis

All experiments were replicated thrice and data were expressed as mean ± standard deviation. All statistical analyses were performed with SPSS 19.0 software. The statistical significance between each group was analyzed using Student’s *t*-test or one-way ANOVA. *P* < 0.05 was considered as statistically significant.

## Results

### Leptin exposure induced EMT in breast cancer cells

**Fig. 1:**
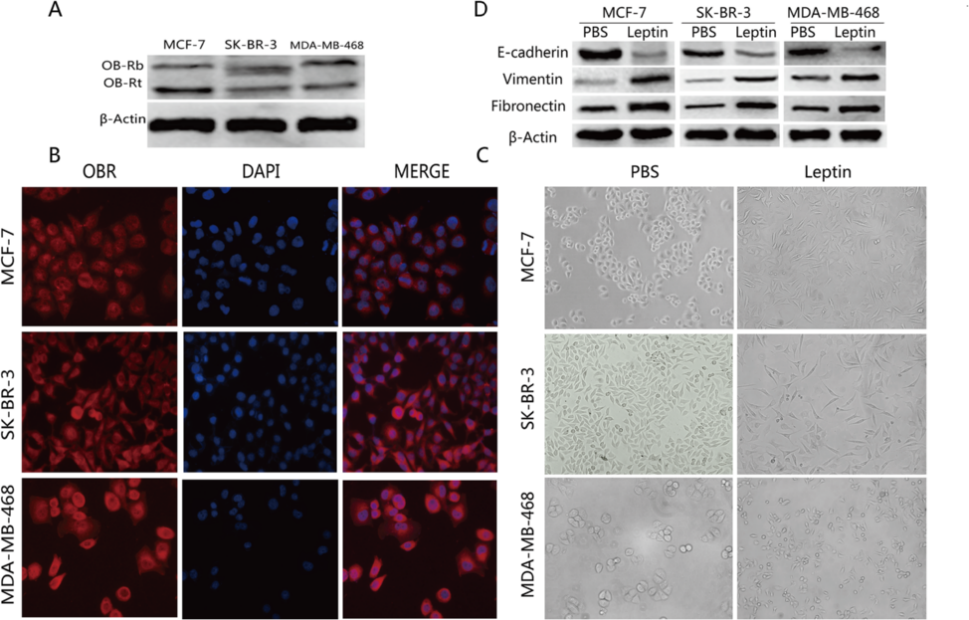
Leptin promoted epithelial-mesenchymal transition in breast cancer cell lines. **a** Western blotting analysis with specific antibodies demonstrated that Ob-Rb and Ob-Rt were expressed in MCF-7, SK-BR-3, and MDA-MB-468 cells. **b** An immunofluorescence assay showed leptin receptor expression on the membrane of breast cancer cells. Original magnification: ×400 **c** Morphological changes of breast cancer cells treated with leptin. The image showed cell-cell junction dissolution and loss of apical-basolateral cell polarity in MCF-7, SK-BR-3, and MDA-MB-468 cells after treatment with 200 ng/mL leptin. Original magnification: ×200 **d** Western blotting analysis demonstrated that leptin treatment upregulated vimentin and fibronectin expression, but downregulated E-cadherin expression in MCF-7, SK-BR-3, and MDA-MB-468 cells. All images were representative examples from three independent experiments

Leptin must bind specific Ob-R receptors to elicit biological functions [10]. The expression of long (Ob-Rb) and short (Ob-Rt) leptin receptor isoforms was evaluated in MCF-7, SK-BR-3 and MDA-MB-468 cells. Western blot analysis indicated that both Ob-Rb and Ob-Rt were expressed in these cells (Fig. 1a), which was also investigated with immunofluorescence (Fig. 1b). In addition, these cell morphological changes such as cell-cell junction dissolution and loss of apical-basolateral cell polarity were revealed after treatment with 200 ng/mL leptin (Fig. 1c). To further demonstrate the expression the mesenchymal markers, vimentin and fibronectin, as well as the epithelial marker, E-cadherin, were assessed. As shown in Fig. 1d, leptin upregulated vimentin and fibronectin expression, while downregulating E-cadherin expression in MCF-7, SK-BR-3, and MDA-MB-468 cells. Totally, these findings suggested that leptin could induce MCF-7, SK-BR-3 and MDA-MB-468 cells EMT.

### Leptin resulted in the upregulation of PKM2 in breast cancer cells

**Fig. 2:**
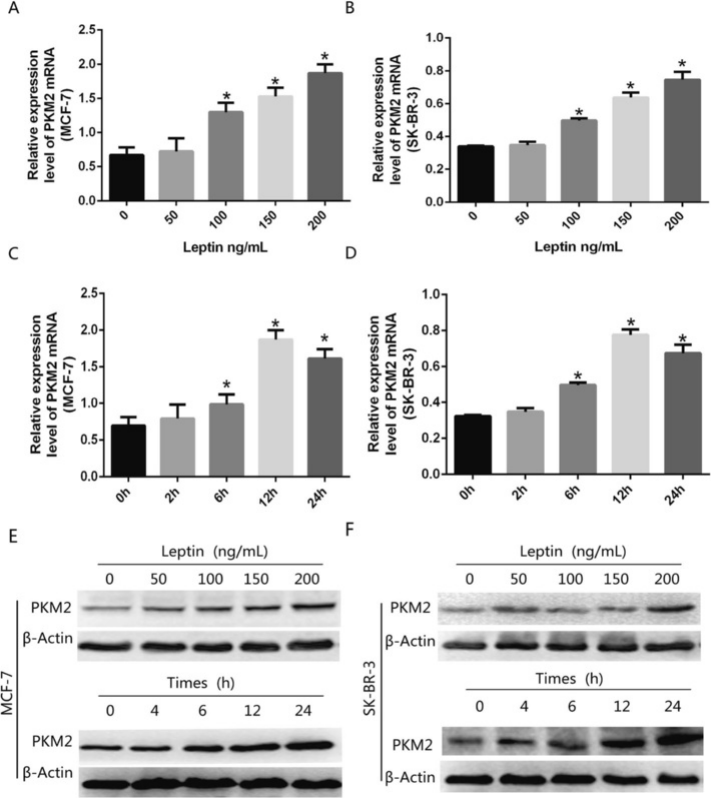
Leptin increased PKM2 expression. (**a**–**f**) Serum-starved MCF-7 and SK-BR-3 cells were exposed to concentrations of leptin ranged from 50 ng/mL to 200 ng/mL or 200 ng/mL leptin for 0 h to 24 h. Q-PCR and Western blotting analysis demonstrated that leptin promoted PKM2 mRNA and protein expression in a dose- and time-dependent manner in MCF-7 and SK-BR-3 cells

Serum-starved MCF-7 and SK-BR-3 cells were exposed to leptin, with the concentration ranging from 50 ng/mL to 200 ng/mL, or 200 ng/mL leptin for 0 h to 24 h. PKM2 mRNA and protein expression were detected by real-time fluorescent quantitative-PCR and Western blot, respectively. It was found that there was a significant increase in PKM2 mRNA and protein expression in a dose- and time-dependent manner (Fig. 2). PKM2 mRNA and protein expression peaked after 200 ng/mL leptin treatment (Fig. 2a, b, e–f) in MCF-7 and SK-BR-3 cells. Therefore, the concentration of leptin at 200 ng/mL was used for the following experiments.

### PKM2 was essential for leptin-induced EMT in MCF-7 and SK-BR-3 cells

**Fig. 3:**
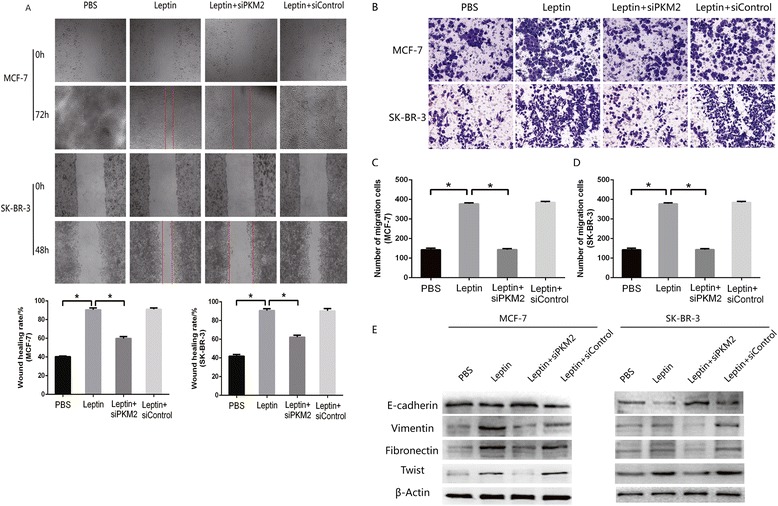
PKM2-siRNA abolished leptin-induced epithelial-mesenchymal transition in breast cancer cells. PKM2-siRNA treatment of MCF-7 and SK-BR-3 cells prior to 200 ng/mL leptin exposure decreased leptin-induced migration (**a**) and invasion (**b**–**d**) (*P* < 0.05). (**e**) PKM2-siRNA abolished leptin-induced downregulation of E-cadherin and upregulation of vimentin, fibronectin and Twist in MCF-7 and SK-BR-3 cells (*P* < 0.05)

To investigate the effects of PKM2 and leptin on the migration and invasion of MCF-7 and SK-BR-3 cells, scratch and invasion assays were performed after treatment with 200 ng/mL leptin and PKM2-siRNA. As shown in Fig. 3a–d, 200 ng/mL leptin markedly promoted the migration and invasion of MCF-7 and SK-BR-3 cells. However, treatment with PKM2-siRNA abolished leptin-induced migration and invasion. Furthermore, the expressions of EMT-associated molecular biomarkers and EMT transcription factor, Twist were also determined. Data showed that leptin upregulated vimentin, fibronectin and Twist expression, but downregulated E-cadherin expression. Notably, this effect was weakened after the addition of PKM2-siRNA (Fig. 3e). These findings indicated that PKM2 had an effect on the upregulation of Twist, and it was also essential for leptin-induced EMT in MCF-7 and SK-BR-3 cells.

### PI3K pathway was involved in leptin-induced EMT and PKM2 expression

**Fig. 4:**
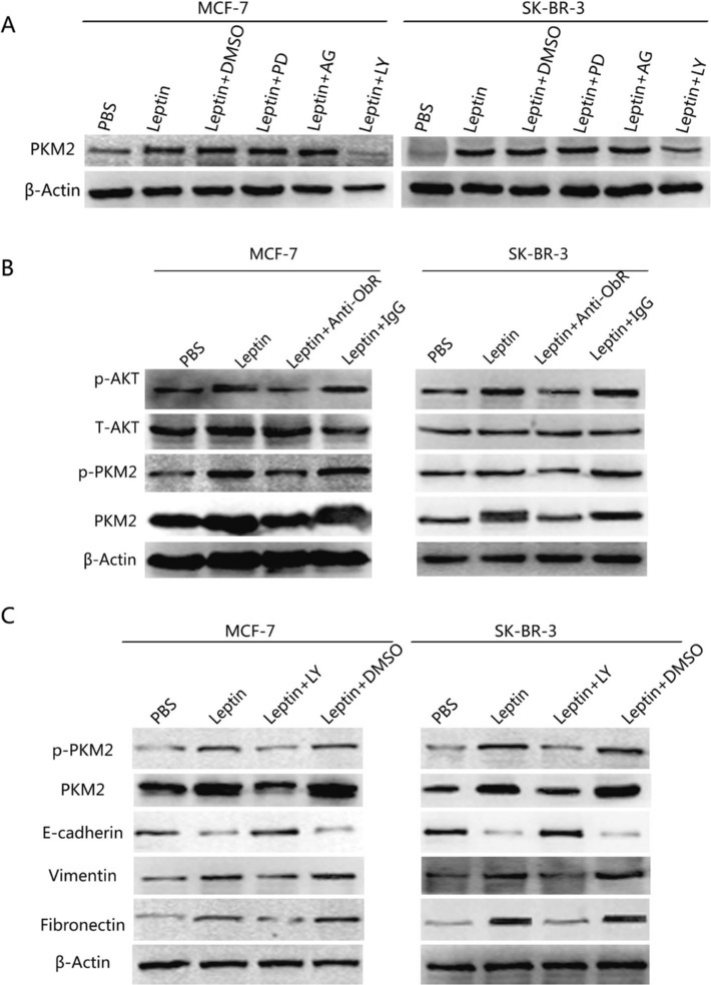
Leptin increased PKM2 expression and induced EMT in breast cancer cells via the activation of PI3K/AKT. **a** Serum-starved MCF-7 and SK-BR-3 cells were treated with PD98059 (MAPK inhibitor, 1.34 μmol/mL), AG490 (STAT3 inhibitor, 5 μmol/mL) and LY294002 (PI3K inhibitor, 10 μmol/mL) for 1 h, followed by 200 ng/mL leptin treatment for 24 h. DMSO was used as a control. Western blotting analysis demonstrated that the PI3K inhibitor LY294002 abolished leptin-mediated increase in PKM2 expression in MCF-7 and SK-BR-3 cells (*P* < 0.05). **b** Antibodies against ObR (4 μg/mL) abolished the leptin-mediated increase in p-AKT, p-PKM2 and PKM2 expression in MCF-7 and SK-BR-3 cells (*P* < 0.05). **c** The PI3K inhibitor LY294002 abolished leptin-mediated downregulation of E-cadherin expression and the upregulation of vimentin, fibronectin, p-PKM2 and PKM2 expression in MCF-7 and SK-BR-3 cells

To explore the signaling pathways involved in leptin-induced breast cancer cells EMT, canonical signaling pathways inhibitors, PD98059, AG490 and LY294002 were used to pretreat serum-starved MCF-7 and SK-BR-3 cells for 1 h, and then treated by 200 ng/mL leptin for 24 h. As shown in Fig. 4a, the leptin-induced upregulation of PKM2 was partially abolished by LY294002 (PI3K/AKT inhibitor), while there was no significant difference when using PD98059 (MAPK inhibitor) or AG490 (STAT3 inhibitor). Besides, the cells were treated with ObR blocking antibodies (4 μg/mL), and then it was found that the expressions of leptin-induced p-AKT, p-PKM2 and PKM2 were abolished by anti-ObR antibodies (Fig. 4b). These findings indicated that PI3K pathway was activated after leptin and ObR binding, which resulted in the overexpression of p-AKT, p-PKM2 and PKM2. Furthermore, EMT biomarker and PKM2 were detected after the addition of LY294002; it was demonstrated that leptin increased vimentin, fibronectin, p-PKM2 and PKM2 expression but downregulated E-cadherin expression, and this effect was reduced by treatment with LY294002 (Fig. 4c). These data indicated that PI3K pathway was involved in leptin-induced EMT and PKM2 expression.

### RNA interference against PKM2 suppressed tumor growth and metastasis in xenograft-bearing mouse models

**Fig. 5:**
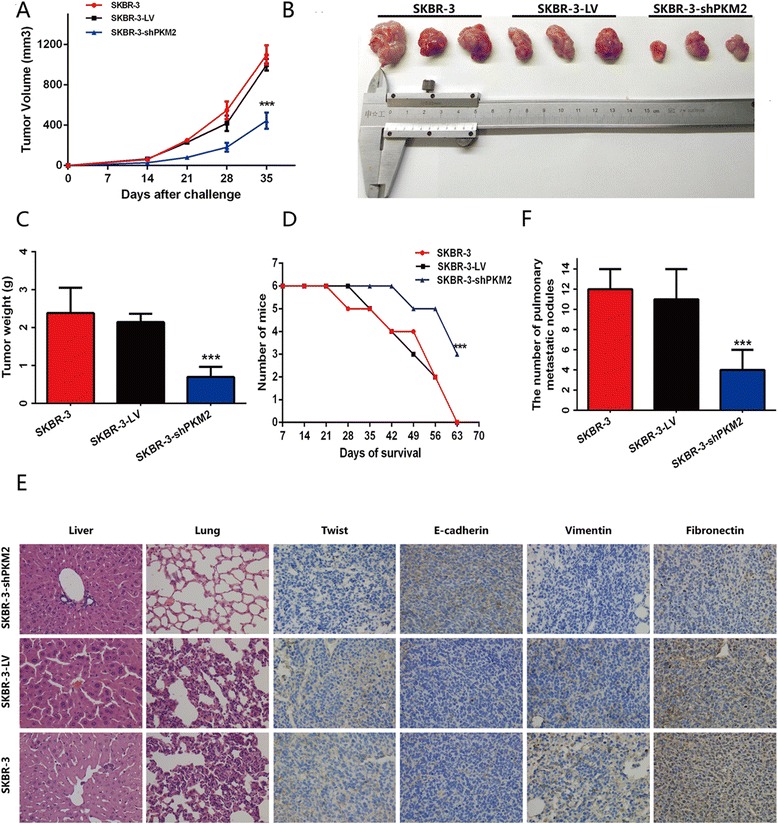
RNA interference against PKM2 inhibited tumor progression in nude mice. SKBR-3, SKBR-3-LV, and SKBR-3-shPKM2 tumor cells (5 × 10^6^ cells/per inoculation) were injected into mammary fat pads of 5-week-old female nude mice (*n* = 9/group). Tumor volumes were measured using calipers and defined as ab^2^/2, where a and b corresponded to length and width, respectively. A cohort of mice was sacrificed 5 weeks after injection. (**a**, **b**, **c**) Tumor volume and primary weights of mice in the SKBR-3-shPKM2 group were significantly smaller than that of mice in the SKBR-3 and SKBR-3-LV groups (*P* < 0.01). (**d**) A survival curve revealed that RNA interference against PKM2 increased the survival of tumor-bearing mice, compared with mice in the SKBR-3 and SKBR-3-LV groups (*P* < 0.01). (**e**–**f**) H&E staining demonstrated that RNA interference against PKM2 inhibited lung metastasis of breast cancer xenografts. Immunohistochemistry staining demonstrated that RNA interference against PKM2 resulted in the increased expression of E-cadherin and decreased expression of vimentin, fibronectin and Twist

To further investigate the role of PKM2 expression in tumor growth in vivo, SKBR-3, SKBR-3-shLV, and SKBR-3-shPKM2 cells were injected into the mammary fat pads of 5-week-old female nude mice. Within 35 days, the average tumor volume of SKBR-3-shPKM2 group was smaller than that of the SKBR-3 and SKBR-3-shLV groups (Fig. 5a and b), and there was no difference in tumor volume between the SKBR-3 and SKBR-3-shLV group. At day 35, mice from each group were sacrificed, and tumor tissue was surgically removed; SKBR-3-shPKM2 treatment resulted in a distinct decrease in tumor size, compared to the size of tumor in the SKBR-3 and SKBR-3-shLV groups (Fig. 5c). In addition, none of the mice survived for 63 days in SKBR-3 and SKBR-3-shLV groups, while 50 % of the mice in SKBR-3-shPKM2 group survived (Fig. 5d). Immunohistochemistry staining was performed to detect E-cadherin, vimentin, fibronectin and Twist expression. As shown in Fig. 5e, the expression of E-cadherin was increased in the SKBR-3-shPKM2 group, while vimentin, fibronectin and Twist expression were decreased, compared with SKBR-3 and SKBR-3-shLV groups. H&E staining showed SKBR-3-shPKM2 resulted in the reduced lung metastases, compared with SKBR-3 and SKBR-3-shLV groups (Fig. 5f). Our data showed that RNA interference against PKM2 suppressed tumor growth and metastasis in xenograft-bearing mouse models.

## Discussion

Epithelial-mesenchymal transition (EMT) has been implicated in tumor cells invasion, metastasis, apoptosis, stemness and treatment failure [26, 27]. Leptin, an established risk factor for many cancers, has been found to act in the cell cycle, proliferation, tumor development, and progression [28–30]. Our previous studies demonstrated that leptin could promote EMT in breast cancer cells [20], and IL-8 was one of the key molecules which affected leptin-mediated EMT. To explore other key regulatory molecules that are involved in leptin-induced EMT of breast cancer cells, gene expression chip array was conducted, which found that a series of enzymes related to glycometabolism increased in breast cancer cells, such as PGK1, PGAM2, PDK2, SUCLG1, DLST, and PKM2. As one of the most increased molecules, PKM2 was verified to increase significantly in MCF-7 and SK-BR-3 cells treated with leptin. Thereby, we hypothesized that leptin promoted epithelial-mesenchymal transition of breast cancer cells via the upregulation of pyruvate kinase M2.

Pyruvate kinase M2 (PKM2), which is a key enzyme of glycometabolism and can act as a transcriptional co-activator, is overexpressed in multiple cancer types and involved in the Warburg effect [31–33]. Studies have shown that PKM2 controls chromosome segregation, cell-cycle progression, cells proliferation, EMT, and tumorigenesis [34–36]. It is firstly revealed in this study that leptin not only induces breast cancer cells EMT, but also upregulates PKM2 expression; however, the underlying mechanism remains unknown.

To this end, firstly, canonical signaling pathways inhibitors, PD98059, AG490 and LY294002, were adopted to explore the signaling pathways involved in leptin-induced breast cancer cells EMT. It was found that LY294002 blocked leptin-induced PKM2, p-PKM2, p-AKT as well as EMT-associated marker expression in MCF-7 and SK-BR-3 cells. As reported in other studies, our results also indicated that PI3K/AKT signaling pathway was involved in EMT [37, 38]. In addition, siPKM2 abolished EMT-associated marker expression and inhibited leptin-induced migration and invasion of those breast cancer cells. So it strongly suggested that leptin could upregulate PKM2 in MCF-7 and SK-BR-3 cells, and PKM2 played a significant role in EMT induction. It is well known that PKM2 also functions as a transcriptional coactivator, and the regulatory mechanism between PI3K/AKT pathway and PKM2 molecular should be elucidated in further researches.

It has been reported that Twist is the key transcriptional factor by down-regulating E-cadherin to promote EMT, cell motility and invasiveness [17, 39]. In addition, increased Twist expression is found in multiple tumors including melanoma [40], prostate [39], pediatric osteosarcoma [41], gastric [42] and breast cancer [17, 43]. The overexpression of Twist positively correlates with tumor aggressiveness and poor prognosis [17, 39, 40]. Therefore, Twist was detected during EMT of MCF-7 and SK-BR-3 cells induced by leptin, and our data showed that leptin upregulated Twist expression, but this effect was weakened by PKM2-siRNA. The potential mechanism in the interaction of PKM2 with Twist needs to be studied.

Xenograft-bearing mouse models showed that RNA interference against PKM2 obviously decreased tumor volume and weight, increased the survival rate of mice, and weakened liver and lung metastases. Besides, the decreased expression of EMT associated markers and Twist were found by IHC staining of tumor tissue.

## Conclusions

This study found that leptin didn’t only induce breast cancer cells EMT, but also upregulated PKM2 expression, which resulted from the activation of PI3K/AKT pathway. Our findings also indicated the crucial role of PKM2 in leptin-mediated EMT, and PKM2 might be one of the key points and potential targets for breast cancer therapy.

## Abbreviations

DMEM: Dulbecco’s modified eagle medium
DMSO: Dimethylsulfoxide
ECL: Enhanced chemiluminescence
EMT: Epithelial-mesenchymal transition
h: Hour
HE: Hematoxylin and eosin
HRP: Horseradish peroxidase
min: Minute
ObR: Leptin receptor
PKM2: M2 type pyruvate kinase
RNA: Ribonucleic acid
shRNA: Short hairpin RNA
